# Fever As the Exclusive Presenting Symptom in a Case of Colon Cancer

**DOI:** 10.7759/cureus.46120

**Published:** 2023-09-28

**Authors:** Mohamad El Labban, Ibtisam Rauf, Mikael Mir, Zhaohui Jin, Syed Anjum Khan

**Affiliations:** 1 Internal Medicine, Mayo Clinic Health System, Mankato, USA; 2 Medical School, St. George's University School of Medicine, True Blue, GRD; 3 Medical School, University of Minnesota Medical School, Minneapolis, USA; 4 Oncology, Mayo Clinic, Rochester, USA; 5 Critical Care Medicine, Mayo Clinic Health System, Mankato, USA

**Keywords:** recurrent fever, organizing abscess, positron emission tomography computed tomography, colon cancer, fever of unknown origin

## Abstract

The definition of fever of unknown origin (FUO) has evolved overtime. Most recently, FUO is recognized as fever with uncertain diagnosis despite three days of hospital admission or three or more outpatient visits. Despite diagnostic medical advancements, FUO remains quite a challenge. In the past, infections, such as abscesses, endocarditis, tuberculosis, and complicated urinary tract infections, were common etiologies of FUO; however, at present, such conditions are readily diagnosed. FUO secondary to malignancy has also been decreasing as a result of radiological advancements. Patients with colon cancer usually present with symptoms secondary to the local anatomy of the tumor. Conversely, fever is an uncommon presentation, especially if it is the sole symptom. Here, we report a unique presentation of colon cancer. Our patient only had intermittent fever for one year before being diagnosed with colon cancer. The fever subsided after resection of the tumor. Despite breakthroughs in diagnostic medicine, FUO remains a challenging diagnosis. Practicing clinicians should have a high level of suspicion to rule out underlying malignancy in the setting of recurrent fevers or FUO.

## Introduction

Fever is a common clinical presentation that can be quite challenging secondary to the wide differential diagnosis. The definition of fever of unknown origin (FUO) has evolved. Petersdorf and Beeson were the first to define FUO in 1961 as a fever higher than 38.3ºC on several occasions, a duration of fever for at least three weeks, and an uncertain diagnosis after one week of study in the hospital [1]. In 1991, Durack et al. redefined FUO as fever with uncertain diagnosis despite three days of hospital admission or three or more outpatient visits [2]. Up to one-third of FUO cases are left undiagnosed [3]. Diagnostic advancements over the past 40 years have reshaped the approach to FUO. Previously associated with classic FUO, infections, such as abscesses, endocarditis, tuberculosis, and complicated urinary tract infections, have been consistently diagnosed. The percentage of undiagnosed cases, although decreased from the 1930s to the 1950s, has been increasing. In fact, as of the early 2000s, undiagnosed FUOs make up more than 50% of the cases [4]. Conversely, FUO, due to malignancy, has decreased over time to an all-time low of 7% of cases in the 2000s [4]. According to the American Cancer Society 2023 statistics, colorectal cancer is the third most common cancer in both males and females each year worldwide, including in the United States. A total of 106,970 new cases of colon cancer and 46,050 new cases of rectal cancer are expected to be diagnosed in 2023 [5]. The presentation of colon cancer is not always straightforward, which can perplex the diagnosis and treatment. Here, we report a case of a 56-year-old male with a one-year presentation of fever with a review of fever as a presenting symptom, specifically in the setting of colon cancer.

## Case presentation

A 56-year-old male patient presented to the urgent care clinic to evaluate recurrent fevers with a one-year duration. The patient is previously healthy and has not undergone any recent health maintenance, including age-appropriate cancer screening. The patient was seen and examined and then referred to the internal medicine clinic. The medical history documented the fever using an oral thermometer (greater than 100.4 F and frequently as high as 107 F). Initially, the fevers occurred once a month; however, the frequency increased to once weekly just before seeking care. Associated symptoms included night sweats and fatigue (only with the fever). The patient denied other associated symptoms, such as weight loss, abdominal pain, change in bowel habits, shortness of breath, cough, chest pain, rash, urinary symptoms, arthralgias, myalgias, oral ulcers, genital ulcers, and focal weakness/numbness. In between the episodes of fever, the patient remained asymptomatic with no limitations to functional status or activities of daily living. There were no recent travels. The patient owned chickens but otherwise denied exposure to other animals. He denied eating or drinking unpasteurized milk products. The patient is a non-smoker, drinks alcohol socially, is monogamous with his female partner, and has never used any illicit drug use, including a history of injections/needle sharing. He had no dental work or other surgical procedures before the fever onset. The patient took no medication, over-the-counter supplements, or herbal tea/drinks. A review of systems was significant for Raynaud's phenomenon for the past 15 years, restricted to his hands and always cold-induced. He denied associated dysphagia, arthralgias, or rash. On exam, the patient was afebrile, well-looking, and had normal vital signs. There was a soft systolic ejection murmur on the second right intercostal space. He otherwise had a normal exam (head, eyes, ears, nose, and throat (HEENT), pulmonary, cardiovascular, abdominal, skin, lymph node, neurological, and musculoskeletal). The initial workup is shown in Table 1.

**Table 1:** Initial workup BUN: blood urea nitrogen; AST: aspartate aminotransferase; ALT: alanine aminotransferase; TSH: thyroid-stimulating hormone; Ab: antibody; HIV: human immunodeficiency virus; Ag: antigen; HCV: hepatitis C virus; IgG: immunoglobulin G; IgM: immunoglobulin M; CRP: C-reactive protein; Scl: scleroderma; RNP: ribonucleoprotein

Lab	Result	Reference
Potassium	3.9	3.6-5.2 mmol/L
Sodium	134	135-145 mmol/L
Chloride	101	98-107 mmol/L
Bicarbonate	28	22-29 mmol/L
BUN	5	8-24 mg/dL
Creatinine	0.79	0.74-1.35 mg/dL
Calcium, total	8.8	8.6 -10.0 mg/dL
Albumin	3.8	3.5-5.0 g/dL L
Protein, total	7.5	6.3 -7.9 g/dL
AST	56	8-48 U/L
Alkaline phosphatase	65	40-129 U/L
ALT	60	7 -55 U/L
Bilirubin, total	0.6	<=1.2 mg/dL
Hemoglobin	13.5	13.2-16.6 g/dL
Hematocrit	39.9	38.3-48.6%
Leukocytes	10.2	3.4-9.6 x10(9)/L
Neutrophils	7.81	1.56-6.45 x10(9)/L
Lymphocytes	0.95	0.95-3.07 x10(9)/L
Monocytes	1.38	0.26-0.81 x10(9)/L
Eosinophils	0.02	0.03-0.48 x10(9)/L
Basophils	0.02	0.01-0.08 x10(9)/L
Platelet count	248	135 -317 x10(9)/L
TSH, sensitive	2.6	0.3-4.2 mIU/L
Rheumatoid factor	16	<15 IU/mL
Antinuclear Ab, HEp-2 substrate	Positive ANA titer: 1:320 ANA pattern: speckled	<1:80 (Negative)
HIV Ag/Ab screen	Negative	
HCV Ab screen	Negative	
Q fever Ab, IgG and IgM, serum	Negative	
CRP	167.3	<=8.0 mg/L
Lactate dehydrogenase	161	122-222 U/L
DNA double-stranded Ab, IgG	2	<=4 (Negative) IU/mL
Cyclic citrullinated peptide Ab	<15.6	<20.0 (Negative) U
Anti-centromere Ab	<1:40	<1:40
Anti-RNA polymerase III	<20	<20 units
Anti-Scl-70 Ab	<20	<20 units
Anti-U1 RNP Ab	<20	<20 units
Anti-U3 RNP (fibrillarin)	Negative	
Anti-Th/To Ab (scleroderma profile)	Negative	
Smooth muscle Ab, IgG, S	<0.2	<1.0 (Negative) U
QuantiFERON-Tb Gold Plus, blood	Negative	

The urine analysis, blood cultures, peripheral blood smear, and chest X-ray were unremarkable. (Red blood cells: red cells were normochromic. Polychromasia, rouleaux formation, schistocytes, and nucleated red blood cells were not identified. White blood cells: Neutrophils showed appropriate segmentation and granulation. Lymphocytes consisted of a heteromorphous population of mature cells. Blasts were not seen on scanning. Platelets: Platelet morphology was unremarkable.) The leukemia/lymphoma immunophenotyping by flow cytometry showed normal immunophenotyping results (no monotypic B-cell population or increase in blasts identified). The patient was then referred to rheumatology for a positive antinuclear antibodies (ANA), positive rheumatoid factor, and history of Raynaud's phenomenon in an otherwise unremarkable evaluation. After the patient was seen by rheumatology, the patient was referred to oncology and infectious disease. Moreover, further blood work was ordered (Table 2).

**Table 2:** Subsequent workup Ab: antibody; GP1: glycoprotein 1; IgA: immunoglobulin A; IgG: immunoglobulin G; RNA: ribonucleic acid; Scl: scleroderma; SS-A: Sjogren syndrome A; SS-B: Sjogren syndrome B;  IgD: immunoglobulin D; RNP: ribonucleoprotein; DRVVT: dilute Russell Viper venom time

Lab	Result	Reference
Myeloperoxidase Ab	<0.2	<0.4 (Negative) U
Proteinase 3 Ab (PR3)	0.3	<0.4 (Negative) U
Beta 2 GP1 Ab IgA	<9.4	<15.0 (Negative) U/mL
Beta 2 GP1 Ab IgG	<9.4	<15.0 (Negative) U/mL
Complement C3	135	75-175 mg/dL L
Complement C4	57	14-40 mg/dL
Cryoglobulin	Negative	Negative %ppt
RNA polymerase III Ab, IgG	15.4	<20.0 (Negative) U
Scl 70 Ab, IgG	<0.2	<1.0 (Negative) U
SS-A/Ro Ab, IgG	<0.2	<1.0 (Negative) U
SS-B/La Ab, IgG	<0.2	<1.0 (Negative) U
IgD	<2	<=10 mg/dL
Complement, total	67	30-75 U/mL
Sedimentation rate	47	0-22 mm/1 h
Ferritin	202	31-409 mcg/L
Creatine kinase	59	39-308 U/L
RNP Ab, IgG	<0.2	<1.0 (Negative) U
Karius test	Negative	
Thrombin time (bovine)	20.0	15.8-24.9 sec
DRVVT mix ratio, DRVVT confirm ratio	1.2 1.05	<1.20 ratio
Staclot delta	5	<=8 sec

**Figure 1:**
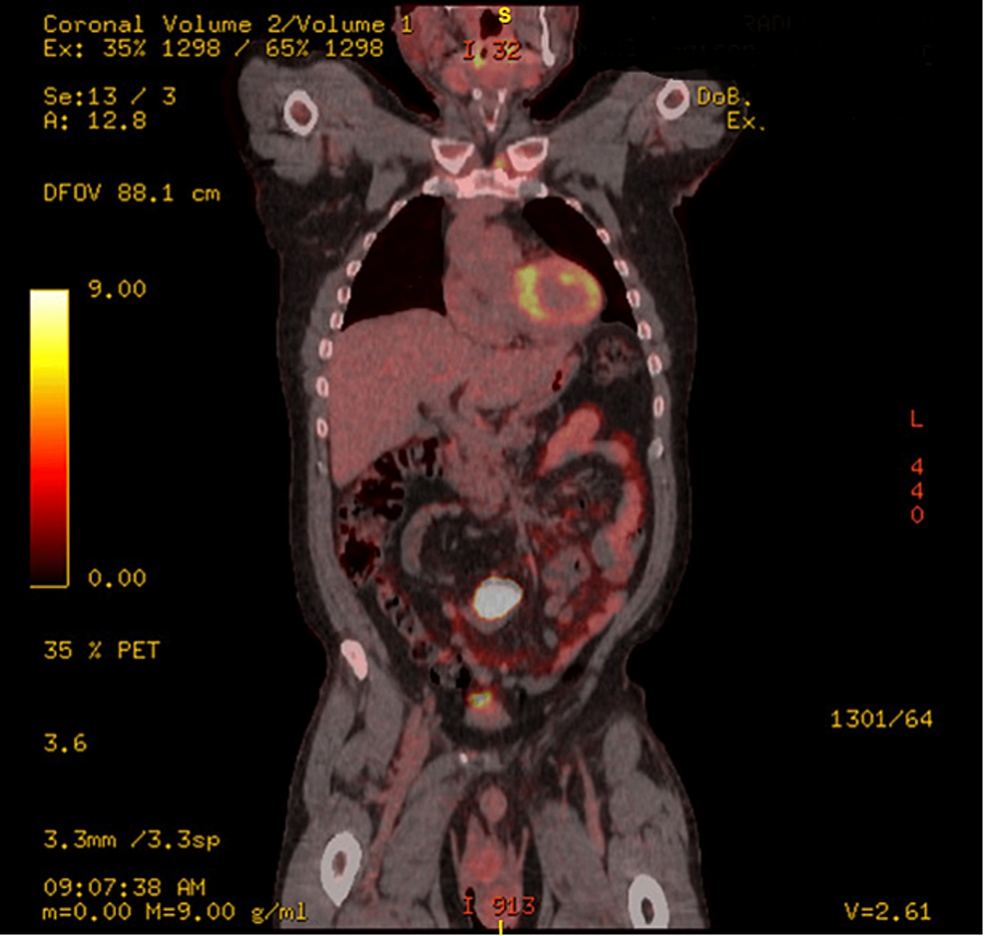
PET scan showing a hypermetabolic mass in the sigmoid colon

After reviewing the workup, there was no obvious answer to the patient's fever. Consequently, it was decided to get a positron emission tomography (PET) scan, which notably showed a highly hypermetabolic mass in the sigmoid colon (Figure 1).

**Figure 2:**
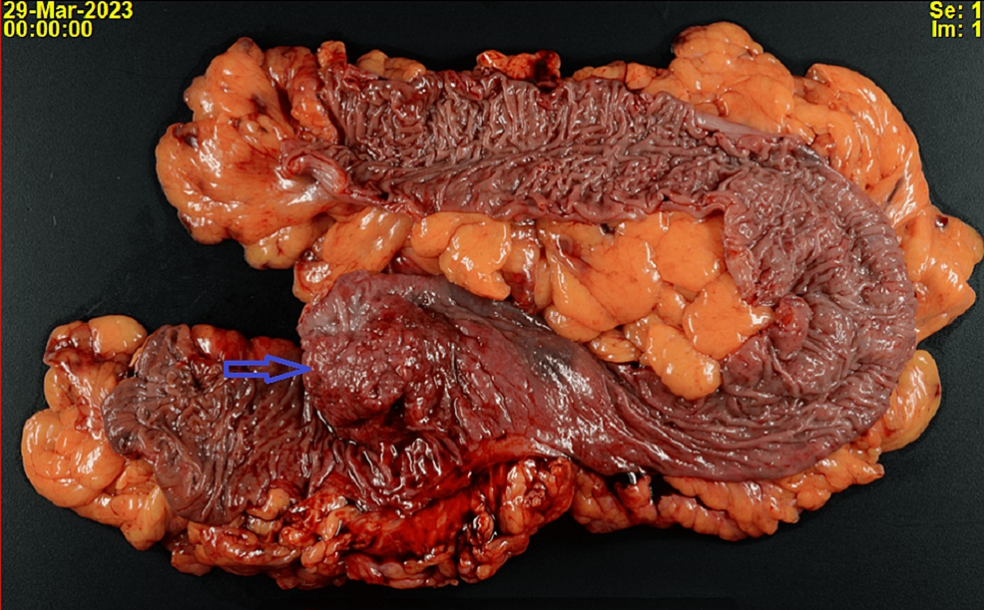
Gross specimen showing a mass in the sigmoid colon

Carcinoembryonic Ag (CEA) level was normal (0.9 ng/mL). The patient then underwent a colonoscopy showing a partially obstructing tumor in the sigmoid colon corresponding to the highly hypermetabolic region on the PET scan. The biopsy was consistent with fragments of tubular adenoma with high-grade dysplasia. Computed tomography (CT) abdomen/pelvis with IV contrast showed a soft tissue sigmoid colon mass at the level of the pelvic brim, with several surrounding involved lymph nodes; otherwise, no abdomen/pelvic evidence of metastatic disease. A colorectal surgeon evaluated the patient, and given the near-obstructing nature of the mass, the patient underwent a robotic sigmoid colectomy with primary anastomosis. Mismatch-repair protein immunohistochemistry showed normal MLH1, MSH2, MSH6, and PMS2 expressions. The final pathology showed focal invasive differentiated adenocarcinoma in a tubulovillous adenoma with high-grade dysplasia and intramucosal carcinoma. Colonic wall with extensive fibrosis and lymphoid tissue with features of organizing abscess is shown in Figure 2). 

All margins and multiple (52) lymph nodes were negative for the tumor. According to the American Joint Committee on Cancer (AJCC) staging system, the cancer was T1N0M0 [6]. On follow-up at the internal medicine clinic three months later, the patient confirmed that the fever did not recur after the resection of the tumor.

## Discussion

The patient was evaluated in three office visits in addition to an extensive workup before establishing a diagnosis and hence met the diagnosis of FUO. Fever is a frequent presentation in patients with solid organ tumors. Our patient's presentation is intriguing because fever was the sole presenting symptom. Among the solid organ tumors, fever is most commonly seen in respiratory tract malignancies [7]. Infectious and non-infectious etiologies can precipitate fever in the setting of active malignancy. In a large prospective study of 477 episodes of fever in cancer patients, Toussaint et al. [7] found that infection was the most common cause of fever (67%), specifically respiratory tract infections followed by bacteremia, urinary tract infections, and soft tissue infections. Non-infectious fever can be caused by the neoplasm (tumor or neoplastic fever), following surgical or other invasive procedures, drugs, and FUO. In contrast to infectious fever, patients with neoplastic fever do not have chills and rigors as often on presentation [8]. The clinical presentation of drug-induced fever is subtle and patients usually look well. Clinical clues include relative bradycardia, rash, elevated eosinophil counts, and transaminitis [9].

Colon cancer can have variable presenting symptoms. Commonly, patients present with symptoms secondary to the local anatomy of the tumor, such as a change in bowel habits, rectal bleeding, abdominal pain, and iron deficiency anemia secondary to occult bleeding [10]. Unusual presentations include malignant fistula formation into adjacent organs, such as the bladder (pneumaturia) and the small bowel and FUO. Around 7% of oncological fever occurs in gastrointestinal malignancies [7]. In a study by Aderka et al., 30% of 92 patients diagnosed with colon cancer had preoperative fever [11]. Fever can be secondary to intra-abdominal, retroperitoneal, and abdominal wall abscesses secondary to localized perforation [12] or *Streptococcus bovis* bacteremia and *Clostridium septicum* sepsis [13]. 

PET scan is a nuclear medicine procedure that measures cellular metabolic activity through fluorodeoxyglucose (FDG) uptake. There has been increasing use of PET-CT in diagnosing FUO. A meta-analysis, based on 1,927 cases, conducted by Kan et al. on the diagnostic potential of PET-CT for the diagnosis of FUO, showed a sensitivity of 84% (95% confidence interval (CI) 0.79-0.89), specificity of 63% (95% CI 0.49-0.75), a positive likelihood ratio of 2.3 (95% CI 1.5-3.4), and a negative likelihood of 0.25 (95% CI 0.16-0.38) [14]. Bharucha et al. showed that the diagnostic yield of PET-CT for malignancy was 95.5% [15]. The PET-CT revealed our patient's diagnosis by showing a hypermetabolic soft tissue mass in the sigmoid colon. 

On follow-up with the internal medicine clinic three months later, the patient mentioned that he was doing well and denied recurrence of fever since the surgery.

## Conclusions

"Humanity has but three great enemies: fever, famine, and war; of these, by far the greatest, by far the most terrible, is fever" (William Osler, 1896). Fever remains one of the most common clinical presentations. A thorough history, physical exam, and workup are crucial to establish a diagnosis. This case report reflects how FUO has changed over time. There has been a decreasing trend for infectious etiologies. Practicing clinicians should have a high level of suspicion to rule out underlying malignancy in recurrent fevers, even as an isolated presenting symptom.
